# Parallel Computing for Quantitative Blood Flow Imaging in Photoacoustic Microscopy

**DOI:** 10.3390/s19184000

**Published:** 2019-09-16

**Authors:** Zhiqiang Xu, Yiming Wang, Naidi Sun, Zhengying Li, Song Hu, Quan Liu

**Affiliations:** 1 School of Information Engineering, Wuhan University of Technology, 122 Luoshi Road, Wuhan 430070, China; xuzhiqiang01@whut.edu.cn (Z.X.); yw6q@virginia.edu (Y.W.); zhyli@whut.edu.cn (Z.L.); 2 Department of Biomedical Engineering, University of Virginia, 415 Lane Road, Charlottesville, VA 22908, USA; ns3gs@virginia.edu

**Keywords:** parallel computing, photoacoustic microscopy, blood flow, correlation analysis, GPU

## Abstract

Photoacoustic microscopy (PAM) is an emerging biomedical imaging technology capable of quantitative measurement of the microvascular blood flow by correlation analysis. However, the computational cost is high, limiting its applications. Here, we report a parallel computation design based on graphics processing unit (GPU) for high-speed quantification of blood flow in PAM. Two strategies were utilized to improve the computational efficiency. First, the correlation method in the algorithm was optimized to avoid redundant computation and a parallel computing structure was designed. Second, the parallel design was realized on GPU and optimized by maximizing the utilization of computing resource in GPU. The detailed timings and speedup for each calculation step were given and the MATLAB and C/C++ code versions based on CPU were presented as a comparison. Full performance test shows that a stable speedup of ~80-fold could be achieved with the same calculation accuracy and the computation time could be reduced from minutes to just several seconds with the imaging size ranging from 1 × 1 mm^2^ to 2 × 2 mm^2^. Our design accelerates PAM-based blood flow measurement and paves the way for real-time PAM imaging and processing by significantly improving the computational efficiency.

## 1. Introduction

Photoacoustic imaging is an emerging imaging modality which shows great potential for basic research and clinical practice [1,2,3,4]. As a hybrid technology, it is based on optical excitation and ultrasonic detection. The pulsed laser light is absorbed by endogenous optical absorbers such as red blood cells (RBCs), and the consequent heat generation induces the emission of acoustic waves as a result of thermoelastic expansion [5]. The photoacoustic signal is then detected by the ultrasonic transducer. Photoacoustic imaging has two major implementations: photoacoustic tomography (PAT) and photoacoustic microscopy (PAM). In PAM, both the optical excitation and ultrasonic detection are focused. Each laser pulse produces a depth-resolved 1-D image (i.e., A-line signal) without mechanical scanning, and 2-D transverse scanning generates a 3-D image [6]. With the unique imaging contrast, PAM has been widely used in biomedical research and proven capable of structural, functional, metabolic and molecular imaging in vivo [7,8,9,10].

Helping to distribute nutrients, oxygen and other products of metabolism, blood flow is essential for tissue viability [11]. Blood flow can be affected by various physiological and pathological factors including metabolic demand and ischemia, and analysis of them benefits the disease diagnosis and treatment [12]. For example, studying the alteration of retinal blood flow may help to identify patients at high risk of cerebrovascular diseases [13].

Correlation analysis of consecutively acquired A-line signals is proven to be an effective method for measuring blood flow by PAM [12,14] and has been successfully applied in many studies [15,16,17]. However, the correlation analysis-based blood flow quantification is time-consuming and thus limits its applications. For example, it takes the conventional CPU-based serial algorithm ~1 h to map the microvascular blood flow over a 5 × 5 mm^2^ region of interest. The computational time can be easily scaled up to days for experiments that consist of multiple groups of such measurements. Moreover, the low computational efficiency of the CPU-based algorithm limits the application of PAM in real-time visualization of rapid hemodynamics [11].

Accelerating data processing with thread-level parallelism, a graphics processing unit (GPU) is frequently used in image processing and computer vision and is specialized for compute-intensive applications [18]. Computer unified device architecture (CUDA) for NVIDIA GPU facilitates the programming and makes GPU one of the most popular acceleration hardware. With these advantages, GPU has been widely adopted to accelerate computation in photoacoustic imaging system, especially in PAT [19,20,21,22,23] for which the image reconstruction algorithm is much more complicated than PAM and needs high performance computation. Currently, several parallel computing methods with GPU have been implemented to reconstruct images in PAT systems such as back-projection (BP)-based PAT [19], finite element method (FEM)-based time-domain quantitative PAT [21] and double-state delay-multiply-and-sum (DS-DMAS)-based PAT [23]. In PAM system, GPUs are mainly adopted for the real-time structure imaging such as displaying maximum amplitude projection (MAP) images of blood vessels in a mouse’s ear [24,25]. High performance computation of quantitative blood flow imaging in PAM has not been reported and remains a challenge.

In this work, we propose a GPU-based parallel computing design for quantitative blood flow imaging in PAM. Two strategies are developed and implemented to accelerate the computational speed. First, the algorithm for blood flow calculation is fully analyzed for optimization and parallel design. The method for correlation analysis is optimized to avoid redundant calculation. Moreover, a computing structure that contains three kinds of parallelism is designed based on the features of each computation step. Second, the parallel design for each computation step is implemented on GPU and optimized by maximizing the utilization of the computing resource, avoiding serial execution on branch divergence and using dedicated (e.g., shared and constant) memory.

The remainder of this paper is organized as follows: measurement principle of blood flow in PAM is introduced in Section 2. The optimization and parallel structure design of the algorithm for blood flow computation is presented in Section 3. In Section 4, the implementation and optimization of the parallel design with GPU is presented; implementations of MATLAB and C/C++ version based on CPU are tested as a comparison. In Section 5, runtimes and blood flow image of final performance tests are given. In Section 6, discussions are made about comparisons with other works, potential limitations and the future research which could be completed based on this study. The conclusion is presented in Section 7.

## 2. Blood Flow Measurement Principle

### 2.1. Imaging System

**Figure 1:**
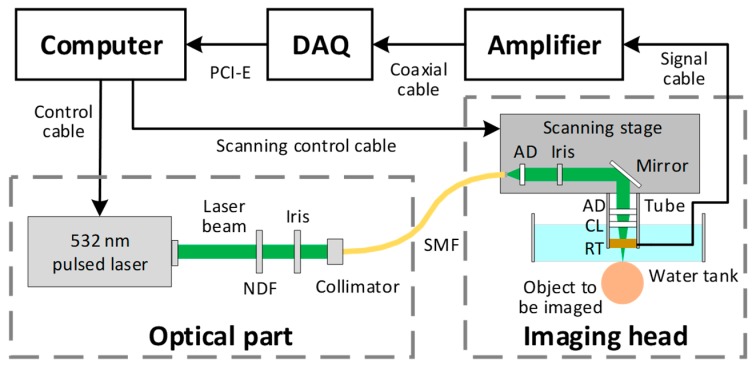
Schematic of the photoacoustic microscopy (PAM) system.

As shown in Figure 1, the PAM system consists of five parts, including the optical part, imaging head, amplifier, data acquisition card (DAQ) and computer system with GPU. In the optical part, the pulsed laser (BX40-2-G, Edgewave, San Diego, CA, USA) is triggered by the computer and outputs nanosecond laser pulses at 532 nm. The beam is attenuated by a neutral-density filter (NDF; NDC-50C-2M, Thorlabs, Newton, NJ, USA) and then reduced to the appropriate diameter by an iris (SM1D12D, Thorlabs, Newton, NJ, USA). The laser beam is coupled into the imaging head through a 2-meter-long single-mode fiber (SMF; P1-460B-FC-2, Thorlabs, Newton, NJ, USA). In the imaging head, the laser beam coming out of the fiber is collimated by an achromatic doublet (AD; AC127-025-A, Thorlabs, Newton, NJ, USA), reshaped by an iris (SM05D5, Thorlabs, Newton, NJ, USA), and focused by an identical doublet through a correction lens (CL; LA1207-A, Thorlabs, Newton, NJ, USA) and the central opening of a customized ring-shaped ultrasonic transducer (RT). Motorized linear scanning stages (PLS-85, PI miCos GmbH, Eschbach, Germany) are used to translate the imaging head for raster scanning of the object to be imaged. A homemade water tank is used to immerse the transducer and the correction lens. A thin layer of ultrasound gel (Aquasonic CLEAR, Parker Laboratories, Newton, NJ, USA) is sandwiched between the object and the transparent polyethylene membrane at the bottom of the water tank for acoustic coupling. The photoacoustic signal is detected by the transducer and then amplified by a commercial low noise amplifier (HD28082, HD Communications Corp, Holbrook, NY, USA). A high-speed DAQ card (ATS9350, AlazarTech, Pointe-Claire, Canada) is used with a sample frequency of 500 MHz, and the sampled data is transferred to a computer though PCI-E interface. The computer is used to synchronize the laser, two-axis linear stages and DAQ during image acquisition to process and display PAM images after the scan.

### 2.2. Measurement Principle

**Figure 2:**
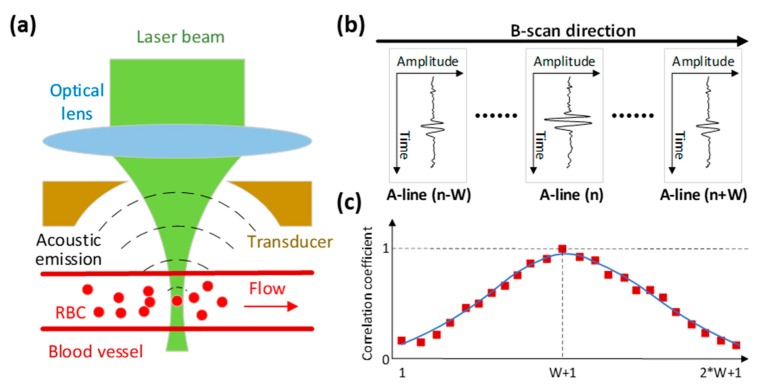
Blood flow measurement principle. (**a**) Experiment configuration. (**b**) Photoacoustic A-lines and B-scan. (**c**) Correlation analysis of the sequentially acquired A-line signals.

Figure 2 illustrates the principle of blood flow measurement in our PAM system. Specifically, RBCs generate photoacoustic waves when they absorb laser energy and undergo a rapid temperature rise. The laser beam used in our system is Gaussian-shaped, which defines the detection volume as shown in Figure 2a. When RBCs move in and out of the volume, the signal fluctuates and is detected by the transducer. The imaging head is mounted on a two-axis motorized stage and a speed of 1 mm/s is set for the cross-sectional scan (i.e., B-scan) in the X direction, during which the laser is triggered at a constant repetition rate to produce A-line signals as shown in Figure 2b. After one B-scan, the imaging head will move one step along the Y direction and then start another B-scan until the entire region of interest is scanned.

In the same B-scan, each specific A-line is respectively correlated with adjacent A-lines to extract the flow-induced temporal decorrelation of the photoacoustic signal as shown in Figure 2c. The number of successive A-lines before and after it is denoted as W. Therefore, the correlation window size including itself is 2 ∗ W + 1. The correlation window is set to be less than 10 ms. Within this time period, the imaging head travels only 10 μm along the B-scan direction, which is comparable to the average diameter of capillary. Linearly proportional to the flow speed, the decay constant of the correlation curve can be used for flow calculation [14]. The correlation analysis allows quantification of blood flow speed at each A-line and eventually pixel-wise flow mapping over the entire scanning area [15].

### 2.3. Animal Experiment

A CD-1 mouse (8-week-old male, Charles River Laboratories) was used for the presented in vivo experiment. A thinned-skull window was created over the region of interest in the mouse brain before the experiment. The temperatures of the water tank and animal body were both maintained at 37 °C throughout the experiment. All experimental procedures were carried out in conformity with the animal protocol approved by the Animal Care and Use Committee at the University of Virginia.

A raw PAM dataset was acquired for quantitative blood flow imaging of a 2 × 2 mm^2^ area in the mouse brain. In the algorithm analysis and implementation test (Section 3 and Section 4), a sub-dataset (imaging size: 1 × 1 mm^2^) of this raw data was used for convenience: (1) it could be applied to evaluate other dataset because the computing complexity for each step in the algorithm is approximately linear to the imaging size; (2) the data size of this sub-dataset was very suitable for buffering in the software could store all the relevant data at once for each test. In the performance test (Section 5), the computational times for sub-datasets with different sizes are compared to get the stable speedup and the MAP and blood flow images of this whole raw dataset are given. During the performance test, when the data size was larger than the buffer size, the input data were divided into several parts and processed sequentially.

## 3. Algorithm Analysis and Parallel Design

### 3.1. Algorithm Analysis

**Figure 3:**
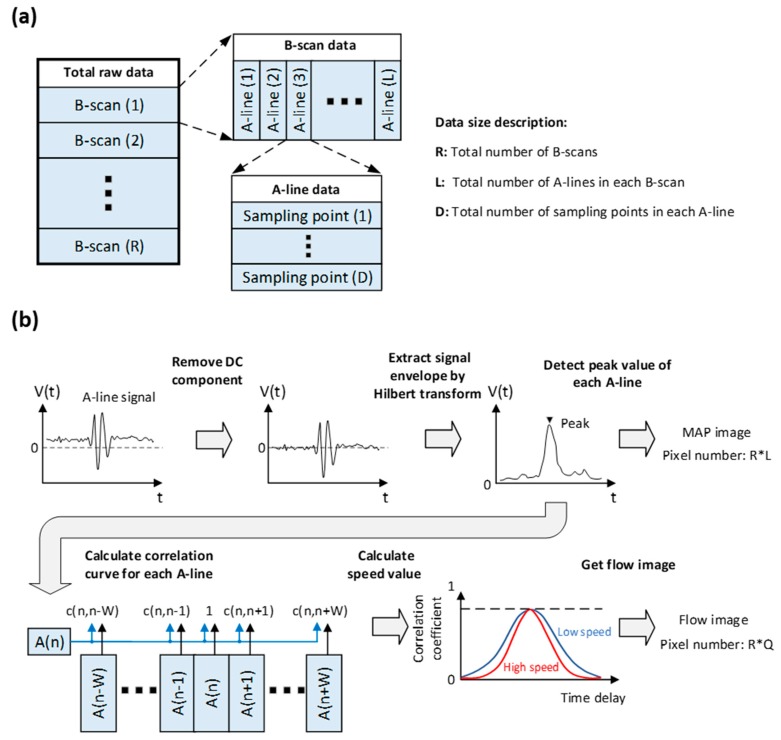
(**a**) Raw data format. (**b**) Flow chart for blood flow calculation.

According to the measurement principle, the algorithm for the flow speed calculation is divided into 7 steps and the major steps are illustrated in Figure 3. In order to demonstrate and compare the amount of data involved in each step of the algorithm, a subset of the raw data with a 1 × 1 mm^2^ size is analyzed as a specific example. The complete computation process is summarized as follows:

1. Read the raw experiment data from the hard disk. As shown in Figure 3a, the number of B-scans in the raw data is marked as R, the number of A-lines in each B-scan is marked as L, and the number of sampling points in each A-line is marked as D, which is even. Each sampling point is a 2-byte integer. For this specific example, R, L and D are 100, 7200 and 512, respectively. Thus, the raw data size is ~737.28 MB.
2. Remove the direct current (DC) component of the raw A-line signal. The DC component is a constant value and remains the same for all the A-line signals. It is obtained before the experiment and subtracted from all sampling points at the beginning of the algorithm. For this specific example, the total number of sampling point is 3.69 × 10^8^.
3. Extract the signal envelope by Hilbert-transforming the A-line signal. Specifically, the original A-line data is firstly transformed by FFT. Then, the Fourier-transformed signal is multiplied by H(n) as follows:
  $$H\left(n\right)=\{\;\begin{array}{c} \;\;\;\;\;1\;\;\;\;\;\;\;\;\;\;\;\;\;\;n=0\;and\;n=D/2\\ \;2\;\;\;\;\;\;\;\;\;\;\;\;\;\;\;\;\;\;\;\;\;0<n<D/2\;\\ \;0\;\;\;\;\;\;\;\;\;\;\;\;\;\;\;\;\;\;\;\;\;D/2<n<D \end{array}.$$
  The multiplied signal is then converted back to the time domain via the inverse-FFT [24]. The total number of A-line is marked as N. For this specific example, N equals 7.2 × 10^5^.
4. Detect the amplitude of the A-line signal. The peak value of the A-line signal is detected to form a MAP image, which is used to show the vascular structure in the region of interest. Each peak value is a 4-byte float data. For this specific example, the data size of the MAP image is ~2.88 MB.
5. Calculate the correlation curve. As shown in Figure 3b, for a specific A-line (denoted as A(n)), the correlation curve that consists of a fixed number of points (denoted as c(j,k)) is obtained by correlating itself with the adjacent A-lines. In a B-scan, the total number of correlation curves is denoted as Q. For the first and last W A-lines of each B-scan, a full correlation curve cannot be obtained. As a result, Q equals L−2∗W. For this specific example, W is set to 23 and the total number of correlation curves is 7.154 × 10^5^.
6. Calculate the flow speed. Least square method is applied to fit the correlation curve and extract the decay constant, from which the flow speed is derived. As shown in Figure 3b, the faster the decay of the correlation curve, the higher the blood flow speed. The decay constant is linearly proportional to the flow speed and the relationship is calibrated with a phantom [15] before the in vivo experiment. After extracting the flow speed value from each of the correlation curve, the blood flow image can be generated. Each flow speed value is a 4-byte float data. For this specific example, the data size of the flow image is ~2.88 MB.
7. Save MAP and flow images to the hard disk.

### 3.2. Optimization of Correlation Curve Calculation

The built-in MATLAB function, *corr*, is utilized to generate the correlation curve in the original algorithm. Each time, one A-line and its adjacent A-lines within the correlation window are put into the function, from which a list of correlation coefficients are returned to generate the correlation curve Curve(n).

**Figure 4:**
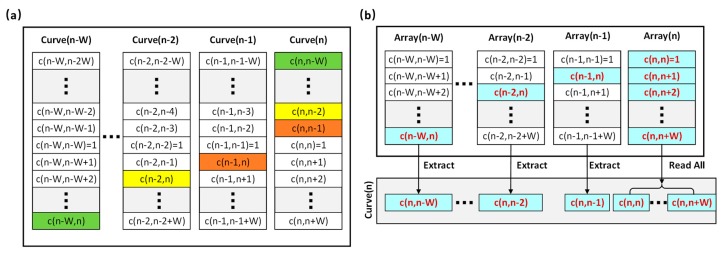
Correlation-computing method in the algorithm. (**a**) Original method. (**b**) Optimized method.

Figure 4a shows a list of correlation curves within one B-scan. In the original algorithm, the total number of computations of correlation (except auto-correlation c(n,n) which equals 1) is denoted as H:

(2) H=R ∗ Q ∗ (2 ∗ W).

For a specific correlation curve Curve(n), redundant calculations occur in the first half of the curve. The values highlighted in color have already been calculated in the previous curve (labeled by the same color).

To avoid the redundant calculation, an optimization method is developed. As shown in Figure 4b, instead of calculating correlation curves pixel by pixel, a two-step process is implemented. First, a correlation coefficient table is computed for all raw data, where each array contains only the second half of the correlation curve. Then, the complete correlation curves are generated by extracting the corresponding values from the table. Similarly, the number of correlation calculation is denoted as H’:

(3) H′=R ∗ Q ∗ W.

With this, the computational time is reduced to half of that in the original method.

### 3.3. Parallel Task Setup

**Figure 5:**
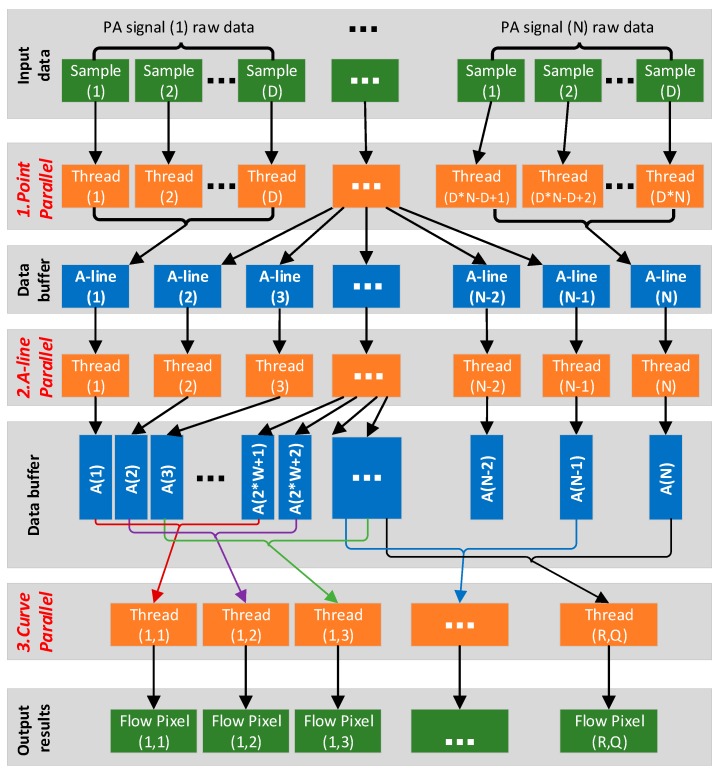
Structure design of parallel computing for the algorithm.

A parallel computing structure containing multiple memory buffers and processing threads in three hierarchies is designed for the algorithm. As shown in Figure 5, from top to bottom, the three hierarchies are point parallel, A-line parallel and curve parallel. Point parallel is a fine-grained design in which each sample point is assigned a thread for calculation. Thus, it is suitable for the step of DC component subtraction. In the A-line parallel, the dataset of each A-line is assigned a thread for the calculation of Hilbert transform and amplitude detection. Curve parallel is a coarse-grained design in which a single thread processes datasets from several A-lines. Thus, it is suitable for the calculation of correlation curve and flow speed value.

## 4. GPU Implementation and optimization

### 4.1. Software and Hardware Platform

The parallel computing structure was implemented with CUDA (version 9.1) based on the GPU hardware. In order to demonstrate the progress, a comparison between the MATLAB (version R2017b), C/C++ and CUDA implementations was made. They were developed under Microsoft Windows 10 Enterprise x64 operating system, and the last two implementations were written in C/C++ under Visual Studio 2015.

The PC for coding and performance test was equipped with Intel Core i7-7800X CPU (3.5 GHz) and 32 GB RAM memory. The CPU is equipped with six cores and supports up to 12 threads working in parallel. NVIDIA GeForce GTX 1080 Ti was chosen for parallel computing. Table 1 lists the specifications.

### 4.2. Initial Implementation

Each calculation step in the blood flow algorithm was implemented with CUDA and evaluated separately according to the parallel design in Figure 5. In the original MATLAB version which has been applied in our previous research [15,16,17], the code had already been optimized by using matrix operations and built-in functions and ran automatically in parallel using multithreads based on CPU. As comparisons, both single-thread and multi-thread C/C++ versions were implemented based on CPU. All the implementations were performed with single precision which was sufficient for the blood flow calculation.

In the implementation test, the sub-dataset analyzed in Section 3 was chosen as the test data. Table 2 shows the runtimes for individual calculation steps, and each runtime was obtained by averaging the outcomes of 10 tests. The acceleration represents the ratio of times between the MATLAB version and the C/C++ or CUDA version.

**Figure 6:**
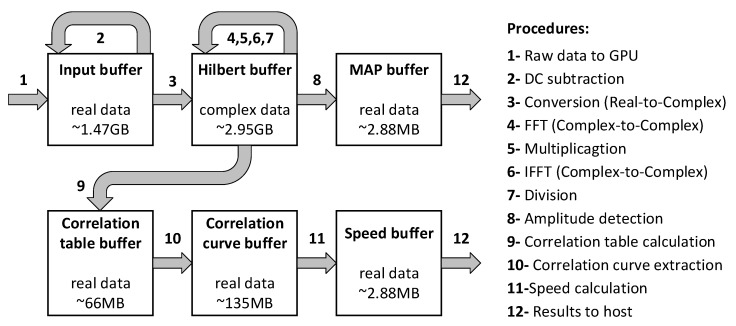
Data flow through the memory buffers in GPU. All the buffers in GPU were allocated with single precision. Procedures from 2 to 11 are kernel functions executed in GPU. Among these kernels, Hilbert transform is completed from 3 to 7 and correlation calculation is completed from 9 to 10.

A custom set of buffers and procedures in GPU were designed as shown in Figure 6. The input PAM data and the complex data after Hilbert transform (stored in input buffer and Hilbert buffer, respectively) occupied most of the memory resource. The intermediate buffers (correlation table and curve buffers) were allocated for the optimized correlation-computation method shown in Figure 4b. Memory size of the results (MAP and speed buffers) were relatively small after the computation.

In GPU processing, the computation time is affected by the number of threads per block. In general, NVIDIA recommends that the number of threads per block should be set to multiples of 64 [24]. After repeated tests, we found that a high computing efficiency could be reached by setting the number of threads per block as the data depth of A-line (i.e., 512 in this implementation).

As shown in Table 2, for the DC subtraction, amplitude detection and speed calculation, the single-thread C/C++ version is a little more efficient than the MATLAB version and a ~10 speedup is acquired by the multi-thread C/C++ version. In the CUDA version, all three steps were implemented with common hardware resource in GPU and remarkable accelerations are achieved.

For the Hilbert transform step, the built-in function, *hilbert*, was called in the implementation with MATLAB, the FFTW library was utilized in the C/C++ code, and the cuFFT library was utilized in the implementation with CUDA. FFTW is a free CPU-based subroutine library for computing the discrete Fourier transform, and its performance is highly optimized. Multi-threaded FFTW was utilized in the multi-thread C/C++ code. The cuFFT is a free library, which is also optimized to provide high performance on NVIDIA GPUs. In the CUDA implementation, the FFT and IFFT were realized by the one-dimensional transform function provided by the cuFFT library, and the number batch for the input parameter was set as the A-line numbers. Execution of FFT and IFFT were paralleled with the GPU hardware resource allocated by the cuFFT library automatically. Results shows that the single-thread C/C++ is slower than the MATLAB version, the multi-thread C/C++ version takes about the same time and a considerable speedup is achieved by the CUDA version.

For the correlation calculation step, the runtime of the single-thread C/C++ version is slightly longer than the MATLAB version and the multi-thread C/C++ takes about the same time. The runtime is reduced to 1856 ms for the initial CUDA version. After adopting the improved algorithm, the runtime is further reduced by half (933 ms), which is in accordance with the theoretical analysis mentioned above.

### 4.3. Optimized Implementation

The computational efficiency of the blood flow algorithm was further improved by maximizing the utilization of the hardware computation resource provided by GPU.

**Figure 7:**
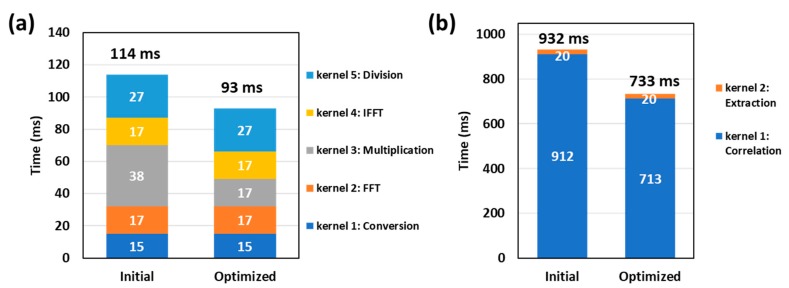
Kernel runtimes. (**a**) Hilbert transformation. (**b**) Correlation calculation.

**Figure 8:**
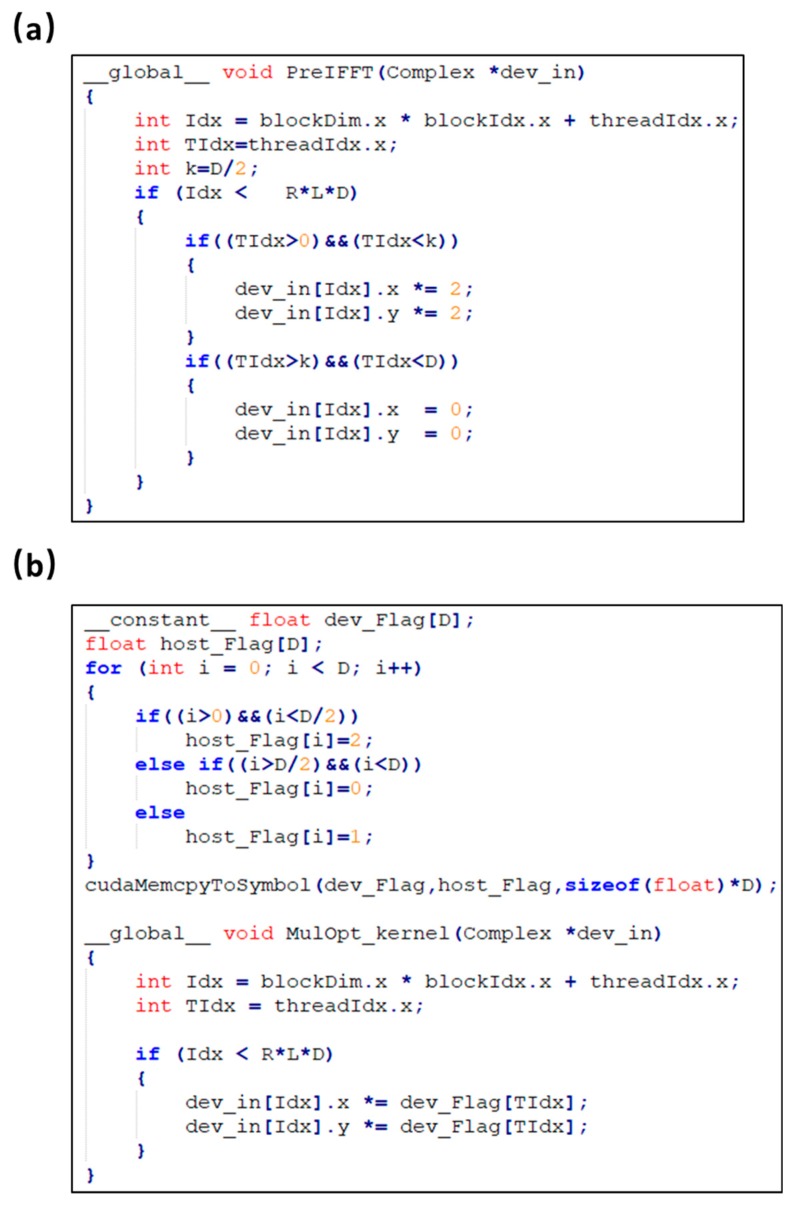
Kernel function of multiplication in Hilbert transformation with CUDA. (**a**) Initial implementation. (**b**) Optimized implementation. Idx, thread address index; TIdx, thread index in a block.

As shown in Figure 7a, five kernels were used in CUDA for the Hilbert transformation. In the initial implementation, the multiplication in kernel 3 contains branch structures (shown in Figure 8a) which impairs the parallelism of threads. As a consequence, the computation efficiency of kernel 3 is the lowest in this step. An optimization was carried out by replacing the branch structure with a lookup table. As shown in Equation (1), the Fourier transformed signal is multiplied by one of three coefficients according to its address index. To avoid the selection, a lookup table that contained the multiplication coefficients (data size: 2 KB) was used. In addition, the constant memory resource in the GPU was used for the lookup table to further improve the efficiency as shown in Figure 8b. A single read operation on the constant memory was broadcast to other near threads, which effectively reduced the memory bandwidth. Our results show that the runtime of kernel 3 is reduced from 38 ms to 17 ms and the acceleration of the Hilbert transformation is increased from 115 times to 140 times.

**Figure 9:**
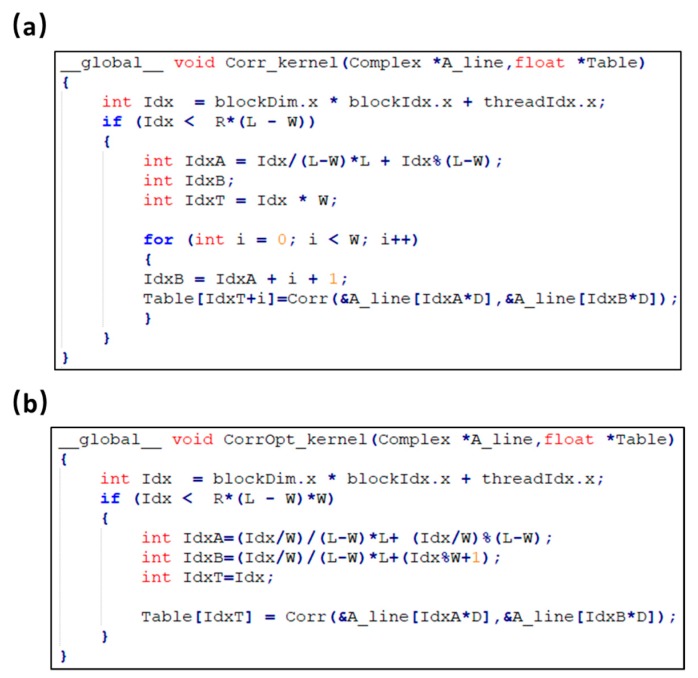
Kernel function of correlation calculation with CUDA. (**a**) Initial implementation. (**b**) Optimized implementation. Idx, thread address index; IdxA and IdxB, the input data addresses for A-line pair; IdxT, the output data address for the correlation coefficient table.

As shown in Figure 7b, two kernels were used for the correlation calculation. In kernel 1, each thread was allocated to calculate a list of correlation coefficients in a loop structure as shown in Figure 9a. Optimization was achieved by replacing the loop structure with a fine-grained parallelism, in which each thread only calculated one correlation coefficient between two A-lines as shown in Figure 9b. Results in Figure 7b show that after the optimization, the runtime of kernel 1 is reduced from 912 ms to 713 ms. The speedup is limited because of the tradeoff between the computing and data access. However, more threads are allocated to accelerate the computing in the optimized CUDA version. The address mapping for the input A-line pair in each thread is complex as shown in Figure 9b, which increases the data access time.

**Figure 10:**
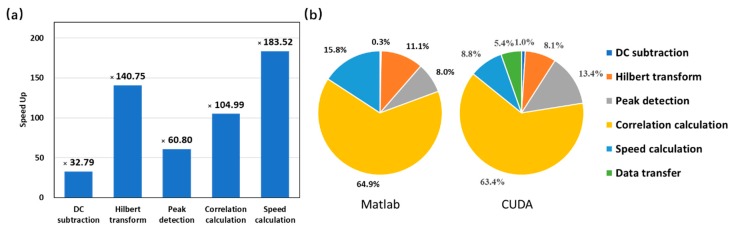
Analysis of the implementation results. (**a**) Speedup of each step. (**b**) Runtime analysis.

The final speedup for each calculation step is shown in Figure 10a. A speedup ranging from 32.79 to 183.52 are achieved for the CUDA implementation compared with the original MATLAB version. Figure 10b shows the percentage of time consumption for each calculation step in MATLAB and CUDA version separately. The correlation calculation step in MATLAB version accounts for over 60% of the entire computational time, and thus its speedup has a great impact on the overall runtime. Although data transfer between CPU and GPU device consumes extra time in the CUDA implementation, it only accounts for a very small portion (~5%).

## 5. Performance Test

**Figure 11:**
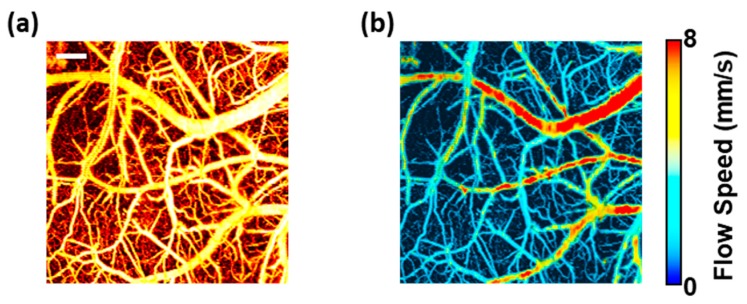
(**a**) Maximum amplitude projection (MAP) image. (**b**) Blood flow image. Scale bar in (**a**) is 250 μm.

A full performance test of this parallel computing design with GPU was implemented with the sub-dataset ranging from 1 × 1 mm^2^ to 2 × 2 mm^2^. Runtime was recorded during the whole computing process (i.e., reading raw data from the hard disk, computing flow speed by the algorithm and writing results back to the hard disk). Table 3 presents the runtime results obtained by averaging the outcomes of ten experiments. Results of the original MATLAB version and the multi-thread C/C++ version based on CPU were listed as a comparison. It shows that a stable acceleration of the parallel computing based on GPU could be realized and the total processing time could be reduced from several minutes to just several seconds when the imaging size ranges from 1 × 1 mm^2^ to 2 × 2 mm^2^. The calculated flow results of MATLAB, C/C++ and CUDA version are essentially the same (the difference is less than 1 × 10^−4^ mm/s, which is orders of magnitude smaller than the microvascular flow in vivo and thus negligible). The MAP and blood flow images computed by CUDA for the total raw dataset (imaging size: 2 × 2 mm^2^) are shown in Figure 11.

## 6. Discussion

Parallel computing based on GPU is proven effective to improve computational efficiency and is extensively adopted in PAT imaging systems, for which the image reconstruction algorithm is usually designed with a high computation complexity to obtain a better image quality [19,20,21,22,23]. In PAM system, GPU has been applied for the real-time structure imaging (MAP) of blood vessels in a mouse’s ear [24,25]. Different from these works, our parallel computation design generates both the structural image (MAP) and functional image (blood flow) and the latter accounts for over 60% of the entire computational time (shown in Figure 10). Similar to [26], we have divided the algorithm into several kernel functions, optimized them separately by maximizing the utilization of computing resource, evaluated their performance and made comparisons between the CPU-based implementations (original MATLAB version and C/C++ version) and the GPU-based implementation (CUDA version). Furthermore, we have put forward a parallel computing structure (shown in Figure 5) containing three kinds of parallelism according to the features of the blood flow imaging algorithm. Therefore, it could be easily updated and applied to other functional imaging like total concentration and oxygen saturation of hemoglobin in PAM [15,16,17].

The parallel computing method proposed in this paper is an off-line computation which only processes data stored in the hardware disk. Further improvements could be made to synchronize the parallel computation with the imaging scanning process in the experiment. Another improvement could be made to optimize the algorithm by computing blood flow just for areas containing vessels instead of all the scanning area. Combing the parallel computation of blood flow imaging proposed in this paper, a real-time high-resolution multiparametric PAM system could be made to visualize the structural and functional dynamics for in vivo experiment.

## 7. Conclusions

We have developed a GPU-based parallel computing method for high-speed quantification of blood flow in PAM. The existing algorithm for blood flow calculation was analyzed and optimized to reduce the computational cost for correlation analysis by half. A parallel computing structure was designed based on the features of the algorithm. The design was implemented and optimized with CUDA based on the GPU hardware platform. A full performance test was implemented, showing that the stable acceleration of the parallel computing design could be realized with the same calculation accuracy and the computation time could be reduced from minutes to just several seconds with the imaging size ranging from 1 × 1 mm^2^ to 2 × 2 mm^2^. Our work accelerates the process of blood flow measurement in PAM and paves the way for real-time PAM imaging and processing by significantly improving the computational efficiency.

## Figures and Tables

**Table 1:** Graphics processing unit (GPU) Specifications.

Parameter	GeForce GTX 1080 Ti
CUDA Architecture	Pascal
CUDA Computer Capability	6.1
Clock Rate (GHz)	1.582
Global Memory (GB)	11
CUDA Cores	3584
Multiprocessor Count	28
SIMD Width	32

**Table 2:** The detailed timings and speedup for each calculation step.

Hardware	CPU					GPU	
Software ^1^	MATLAB Time (ms)	Single-Thread C/C++		Multi-Thread C/C++		CUDA	
		Time (ms)	Speedup	Time (ms)	Speedup	Time (ms)	Speedup
Data to GPU	—	—	—	—	—	60	—
DC subtraction	371	134	×2.77	37	×10.02	11	×33
Hilbert transform	13,131	19,760	×0.66	10,978	×1.19	114	×115
Amplitude detection	9456	3877	×2.44	732	×12.92	156	×61
Correlation calculation	76,985	91,037	×0.85	71,683	×1.07	1856	×41
						933 (new ^2^)	×83
Speed calculation	18,740	5894	×3.18	1734	×10.81	272	×68
Data to host	—	—	—	—	—	2	—

^1^ All the software were executed with single precision. Twelve threads supported by CPU were used for the MATLAB and Multi-thread C/C++ version. All the speedup values were calculated based on the MATLAB version. ^2^ Implemented with the new method shown in Figure 4b.

**Table 3:** Performance Test.

Image Size (mm^2^)		1	1.5	2	2.5	3	3.5	4
A-line number (×10^6^)		0.72	1.08	1.44	1.8	2.16	2.52	2.88
Data size (GB)		0.74	1.11	1.47	1.84	2.21	2.58	2.95
MATLAB ^1^ Runtime (s)		123.63	201.37	263	327.68	388.02	460.7	528.03
Multi-thread C/C++	Runtime (s)	86.16	124.52	168.99	213.46	260.6	305.07	350.43
	Speedup ^3^	×1.43	×1.62	×1.56	×1.54	×1.49	×1.51	×1.51
CUDA ^2^	Runtime (s)	1.52	2.47	3.34	4.08	5.03	5.72	6.59
	Speedup	×81.34	×81.52	×78.74	×80.31	×77.14	×80.54	×80.13

^1^ All the software versions were executed with single precision. Twelve threads supported by CPU were used for the MATLAB and Multi-thread C/C++ version. ^2^ Implemented on GPU with multiple threads. ^3^ All the speedup values were calculated based on the MATLAB version.
